# Murine Chronic Pancreatitis Model Induced by Partial Ligation of the Pancreatic Duct Encapsulates the Profile of Macrophage in Human Chronic Pancreatitis

**DOI:** 10.3389/fimmu.2022.840887

**Published:** 2022-04-01

**Authors:** Cheng Peng, Guangping Tu, Li Yu, Peng Wu, Xianlin Zhang, Zheng Li, Zhiqiang Li, Xiao Yu

**Affiliations:** ^1^ Department of Hepatopancreatobiliary Surgery, Third Xiangya Hospital, Central South University, Changsha, China; ^2^ Department of Radiology, Third Xiangya Hospital, Central South University, Changsha, China; ^3^ Department of General Surgery, Renhe Hospital, Three Gorges University, Yichang, China

**Keywords:** pancreatitis, animal model, macrophage, cytokines, pyroptosis

## Abstract

Immune responses are an integral part of the pathogenesis of pancreatitis. Studies applying the mouse model of pancreatitis induced by partial ligation of the pancreatic duct to explore the pancreatic immune microenvironment are still lacking. The aim of the present study is to explore the macrophage profile and associated regulatory mechanisms in mouse pancreatitis, as well as the correlation with human chronic pancreatitis (CP). In the present study, the mouse model of pancreatitis was induced by partial ligation of the pancreatic duct. Mice in the acute phase were sacrificed at 0, 4, 8, 16, 32, 72 h after ligation, while mice in the chronic phase were sacrificed at 7, 14, 21, 28 days after ligation. We found that the pancreatic pathological score, expression of TNF-α and IL-6 were elevated over time and peaked at 72h in the acute phase, while in the chronic phase, the degree of pancreatic fibrosis peaked at day 21 after ligation. Pancreatic M1 macrophages and pyroptotic macrophages showed a decreasing trend over time, whereas M2 macrophages gradually rose and peaked at day 21. IL-4 is involved in the development of CP and is mainly derived from pancreatic stellate cells (PSCs). The murine pancreatitis model constructed by partial ligation of the pancreatic duct, especially the CP model, can ideally simulate human CP caused by obstructive etiologies in terms of morphological alterations and immune microenvironment characteristics.

## Introduction

Pancreatitis mainly includes acute and chronic pancreatitis. Acute pancreatitis (AP) is a disease characterized by acinar cell death and local and systemic inflammation (1, 2), with a global incidence of 34 cases per 100,000 persons (3). Chronic pancreatitis (CP) is a progressive inflammatory disease of the pancreas characterized by irreversible morphological changes, progressive fibrosis, as well as loss of exocrine and endocrine functions (4, 5). Epidemiological studies have shown that the incidence of CP in the population ranges from 4.4 to 14 cases per 100,000 people (6, 7). Patients suffering from pancreatitis often have a great physical and mental burden, and research on pancreatitis remains a huge challenge (8).

The etiology of pancreatitis mainly includes metabolic toxicity such as alcoholism and pancreaticobiliary obstructive disease such as pancreatic duct gallstones. In addition, a small part of pancreatitis is caused by autoimmune and genetic factors (1, 4, 9). Immune responses are an integral part of the pathogenesis of pancreatitis, determining its development and outcome (10). However, studies on the pancreatic immune microenvironment in pancreatitis are limited due to the paucity of pathological specimens from patients with pancreatitis. Therefore, an animal model that can better mimic clinical pancreatitis is of great value for the progress of pancreatitis research. Repeated multiple injections of cerulein in mice are the current mainstream protocol of constructing AP and CP, however, the significant drawback is that this model cannot mimic the clinicopathological features of any type of pancreatitis (11, 12). In 2015, Sender and colleagues first developed a mouse model of pancreatitis that more closely matched the clinicopathological features by partial ligation of the pancreatic duct (13). Duct dilation after ligation mimicking the state of increased pressure in the pancreatic duct from causes such as pancreatic duct stones, intense inflammatory response, and tissue regeneration (13). However, studies applying this model to explore the pancreatic immune microenvironment in pancreatitis are still lacking.

Macrophages are the major innate immune cell subset characterized by heterogeneity and plasticity (14, 15). Activated macrophages are divided into M1 and M2 subtypes, both M1 and M2 macrophages are closely related to the inflammatory response, where M1 macrophages are mainly involved in the pro-inflammatory response by producing pro-inflammatory mediators such as interleukin (IL)-1β, IL-6, IL-12, and tumor necrosis factor (TNF-α). On the contrary, M2 macrophages are mainly involved in the anti-inflammatory response and tissue regeneration process by secreting IL-4, IL-10, IL-13, and transforming growth factor-β (TGF-β) (16, 17). Studies have shown that macrophages are involved in the development of cerulein-induced AP (18, 19) and CP (20, 21) in mice. However, the dynamics of macrophages and the associated regulatory mechanisms remain unclear in mouse models of pancreatitis induced by partial ligation of the pancreatic duct, as well as in human CP.

In the present study, we revealed the dynamic changes of macrophages in the pancreatitis of mice constructed by partial ligation of the pancreatic duct, including the acute and chronic phases. The progression of pancreatitis from the acute to the chronic phase is also accompanied by a dynamic shift of macrophages from the M1 to the M2 subtype. IL-4 is involved in the development of CP and its main source is pancreatic stellate cells (PSCs) rather than helper T cells (Th cells), which may be an important reason for the polarization of macrophages to the M2 subtype. The murine AP model constructed by partial ligation of the pancreatic duct exhibited more severe pathological alterations with higher necrosis and hemorrhage scores, which is more consistent with the clinically severe acute pancreatitis (SAP) than the cerulein induced model. Moreover, the murine CP model constructed by this method is highly consistent with human CP caused by obstructive etiologies in terms of pathological morphology and pancreatic immune microenvironment and is therefore well suited for the study of CP.

## Materials and Methods

### Reagents

Cerulein(HY-A0190) was purchased from MedChemExpress (USA). Primary antibodies: IL-6 (GB11117), TNF-α (GB11188) were purchased from Servicebio, Inc(Wuhan, China). α-SMA (GB111364), CD68 (GB14043), F4/80 (GB11027) were purchased from Servicebio, Inc. IL-4 (AF5142) and Cleaved Caspase1 (AF4022) were purchased from Affinity Biosciences (Jiangsu, China). CD206 (60143-1-IG) was purchased from Proteintech Group, Inc (Wuhan, China). Inos (13120) was purchased from Cell Signaling Technology(Shanghai, China), CD4 (SC-13573) was purchased from Santa Cruz Biotechnology Co., Ltd (Shanghai, China). Secondary antibodies: HRP-goat Anti-Rabbit IgG (H+L) (GB23303), Cy3 conjugated Goat Anti-Rabbit IgG (H+L)(GB21303), FITC conjugated Donkey Anti-Rabbit IgG (H+L)(GB22403), FITC conjugated Goat Anti-Mouse IgG (H+L)(GB22301) were purchased from Servicebio, Inc. Anti-mouse IgG (H+L), F(AB ‘)2 Fragment (Alexa Fluor^®^ 594 Conjugate) (8890) and Anti-rat IgG (H+L), (Alexa Fluor^®^ 488 Conjugate) were purchased from Cell Signaling Technology.

### Animals

Mice were housed in a specific pathogen-free (SPF) facility. Breeding pairs of C57BL/6J mice (8-10 weeks) were purchased from Hunan SJA Laboratory Animal Co., Ltd. All mice used in the study were male and 6-8weeks old and were acclimatized for one week prior to the formal experiment. Mice were housed in a 12-hour alternating day and night environment and fed with standard laboratory chow and water ad libitum. Animal care and use were approved by Central South University institutional animal care and use committees (NO. 2019SYDW0125).

### Induction of Pancreatitis in Mice

**Figure 1:**
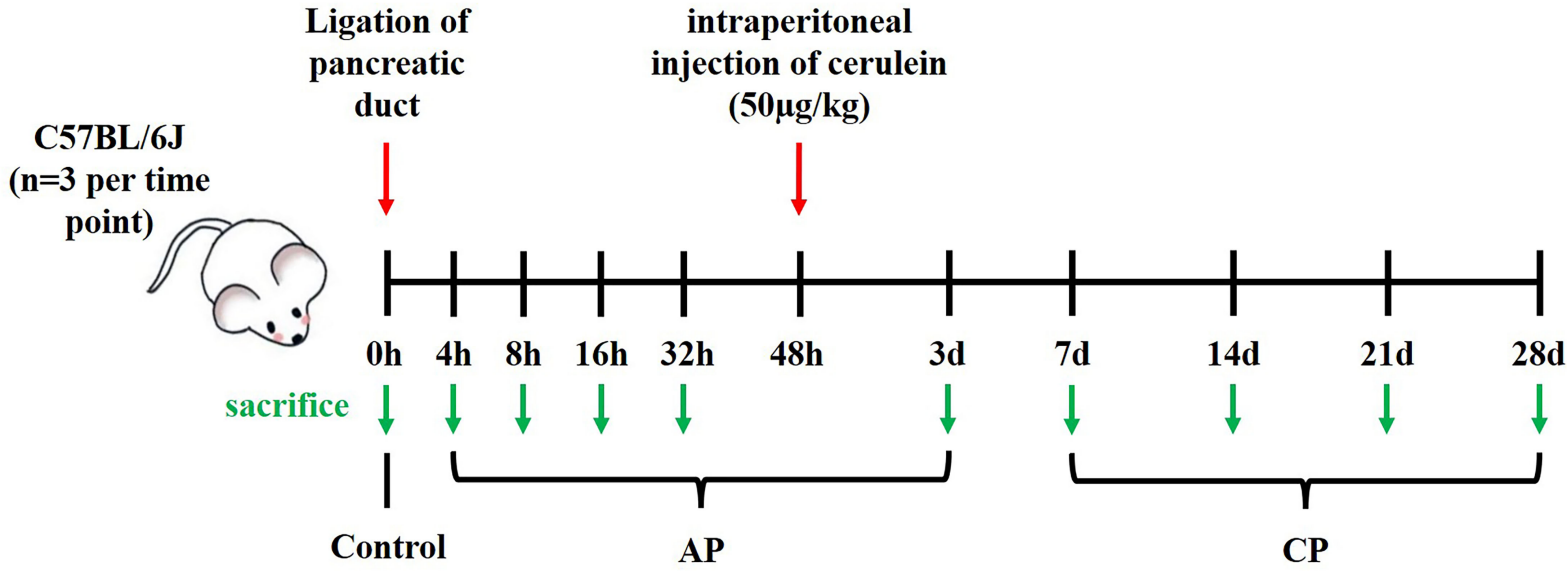
Schematic diagram for constructing a mouse model of pancreatitis induced by partial ligation of the pancreatic duct.

Pancreatitis was induced by partial ligation of the pancreatic duct at the junction of the pancreatic gastric, splenic and duodenal lobes, just below the pylorus of the stomach (**Supplementary Figure 1**), and received a single intraperitoneal injection of cerulein (50µg/kg/body weight) 48 hours after surgery. The mice were sacrificed 3, 7, 14, 21, 28 days after ligation. Mice sacrificed at day 3 were used as AP model, while those sacrificed at day 7, 14, 21 and 28 were served as CP model, as described previously (13, 22). In addition, we also constructed a group of AP mouse models that underwent partial ligation of the pancreatic duct without caerulein injection. This group of mice were sacrificed 0, 4, 8, 16 and 32 hours after ligation. Three mice were used in each time point. A schematic diagram for constructing the pancreatitis model is shown in **Figure 1**. Pancreatic tissue from sacrificed animals was immediately fixed with 4% paraformaldehyde for histological examination.

### Serum Amylase Measurement

Blood samples were stored at 4°C overnight and then centrifuged at a speed of 3000 rpm for 10 min. Serum was collected as supernatant. Amylase levels were detected using the fully automatic biochemistry analyzer with standard techniques (Chemray 240, Redu Life Technologies, Shenzhen, China).

### Histological Assessment

Mice were euthanized by inhaling excessive CO_2_ after designated treatment. Specimens were fixed with 4% paraformaldehyde and embedded in paraffin. Continuous sections of pancreas (3μm) were stained with hematoxylin and eosin (H&E) for histopathological analysis. Five random high-power fields were selected for each HE slice and scored individually. The histopathology of the AP injury was evaluated according to the criteria proposed by Schmidt et al (23), which was based on edema, necrosis, inflammation and hemorrhage. Severity of the CP was graded by a scoring system proposed by Demol et al. (24), which was based on abnormal architecture, glandular atrophy, fibrosis, and pseudotubular complexes. The final score is expressed as the average of these values.

Immunohistochemistry assays were carried out on mice pancreatic tissues to detect and score TNF-α and IL-6 expression. The immunohistochemistry assay was based on the horseradish peroxidase system. Positive staining area was observed and separately evaluated by two experienced pathologists. Mean optical density (IOD) was measured by Image Pro Plus 6.0.

### Immunofluorescence

The paraffin sections were dewaxed to water, and the tissue sections were placed in a repair box for antigen repair. Bovine serum albumin (BSA) was dropped for blocking. Sections were incubated overnight with primary antibodies at 4°C. Then, sections were incubated with secondary antibodies for 1 hour at room temperature. Nuclear staining was performed with DAPI, and the spontaneous fluorescence quenching agent was added. For primary antibodies of the same species origin, we used a Tyramide signal amplification kit (G1235-100T, Servicebio, Wuhan, China) for fluorescent double-label staining. The fluorescence images were captured by a fluorescent microscope. The number of F4/80+iNOS+, F4/80+CD206+, F4/80+Cleaved Caspase1+, CD68+iNOS+, CD68+CD206+, CD68+Cleaved Caspase1+, α-SMA+IL-4+ or CD4+IL-4+ cells as well as the mean fluorescence intensity (MFI) of α-SMA or IL-4 were calculated by Image J 1.52.

### Sirius Red and Masson Staining

The collagen content of lesions was assessed using Sirius Red staining kit (G1018, Servicebio, Wuhan, China) and Masson staining kit (G1006, Servicebio, Wuhan, China) according to the manufacturer’s instructions. In brief, the paraffin tissue sections were deparaffinized with xylene and rehydrated in serial dilutions of ethanol. For Sirius Red staining, the sections were stained with Sirius Red solution, then dehydrated quickly and seal with neutral gum. For Masson staining, the sections were stained sequentially with Masson A, B, C, D, E and F solution following the instruction of the manufacturer’s protocol, then differentiation using 1% glacial acetic acid, finally dehydrated quickly and seal with neutral gum. Collagen fibers appeared red in Sirius Red staining and blue in Masson staining. The percentage of fibrosis area in Masson staining was measured by Image J 1.52.

### Human Pancreatic Samples

Human pancreatic tissue samples of three CP patients with obstructive etiologies (1 periampullary tumor, 1 obstructive gallstone and 1 main duct pancreatic stones) (25) and normal pancreas of three nonmalignant pancreatic patients (1 intraductal papillary mucinous neoplasm, 1 pancreatic lymphangioma and 1 pancreatic serous cystadenoma) undergoing surgery were obtained from the Department of Hepatobiliary and pancreatic Surgery, Third Xiangya Hospital, Central South University, with the approval of the Ethics Committee of the Third Xiangya Hospital of Central South University (NO. 21175) and the consent of patients. No morphological abnormalities were found in normal pancreatic samples from nonmalignant pancreatic patients, and no visible tumors were found in samples from patients with CP. Detailed clinical characteristics of each patient are provided in **Table 1**.

**Table 1:** Clinical characteristics of human pancreatic biopsies.

No.	Age (y)	Sex	Duration	Etiology	Pancreatic insufficiency	Dilation of main pancreatic duct	Group
**1**	67	Male	6 months	Intraductal papillary mucinous neoplasm	Absent	Absent	Nornal tissue
**2**	60	Female	2 years	Pancreatic lymphangioma	Absent	Absent	Nornal tissue
**3**	63	Female	6 months	Pancreatic serous cystadenoma	Absent	Absent	Nornal tissue
**4**	42	Male	2 years	Periampullary tumor	diabetes mellitus	7mm	Chronic pancreatitis
**5**	49	Male	6 years	Obstructive gallstone	dyspepsia	6mm	Chronic pancreatitis
**6**	54	Male	3 years	Main duct pancreatic stones	diabetes mellitus	5mm	Chronic pancreatitis

### Statistical Analysis

All data are expressed as the mean ± standard error of the mean of at least three animals or experiments. Data analysis was performed using GraphPad Prism 8.1 software. Comparisons between two experimental groups were performed by a 2-tailed Student’s t-test, and multiple comparisons were performed using the one-way ANOVA statistical test followed by Dunnett test. A statistically significant difference was considered when P < 0.05.

## Results

### AP Induced by Partial Ligation of Pancreatic Duct Presented as Necrotizing Pancreatitis

**Figure 2:**
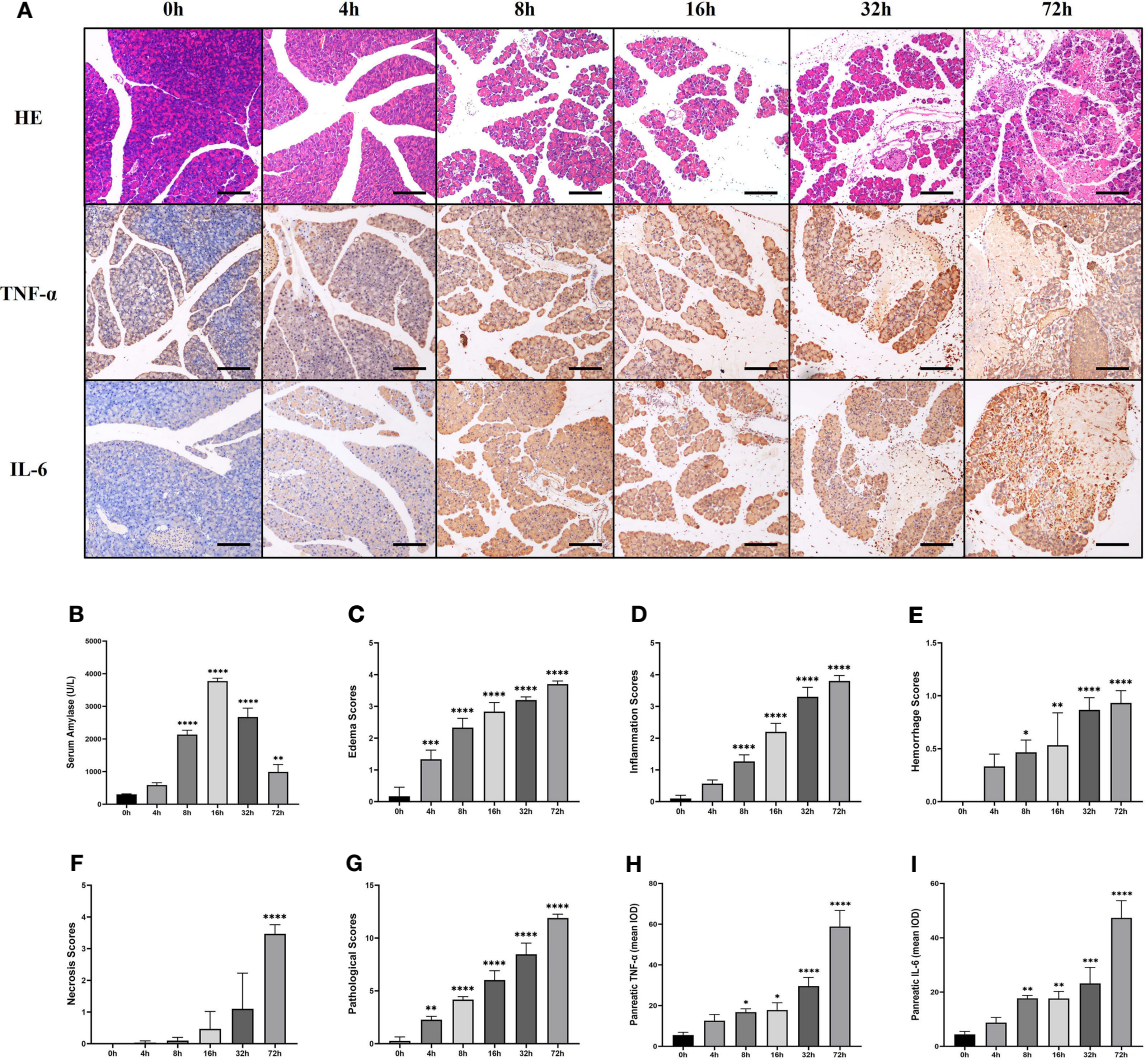
The pancreas of mice presented significant lesions after ligation of pancreatic duct. Serum amylase level started to increase after 4h, peaked at 16h, and then gradually decreased. Pancreatic edema score, inflammation score, hemorrhage score, necrosis score as well as total score, and the expression of TNF-α and IL-6 were elevated over time and peaked at 72h. **(A)** HE staining and immunohistochemical staining of TNF-α and IL-6 of pancreas in mice. **(B)** Serum amylase. **(C)** Pancreatic edema score. **(D)** Pancreatic hemorrhage score. **(E)** Pancreatic hemorrhage scores. **(F)** Pancreatic necrosis scores. **(G)** Total pathological scores. **(H)** Mean IOD of pancreatic TNF-α. **(I)** Mean IOD of pancreatic IL-6. Values are shown as means ± SE. n = 3 per time point. *P < 0.05, **P < 0.01, ***P < 0.001, ****P < 0.0001 versus 0h. Scale bar=150μm. IOD, integrated optical density.

The serum amylase level in AP mice induced by partial ligation of the pancreatic duct started to increase after 4h, peaked at 16h, and then gradually decreased (**Figure 2B**). We explored the pathological changes of the pancreas within 72h after partial ligation of the pancreatic duct, and the results showed that the pancreatic lesions in the AP model constructed by this method were severe, with massive necrosis of pancreatic acinar cells, interstitial edema, parenchymal loss, hemorrhage, and inflammatory cell infiltration starting gradually at 4h (**Figure 2A**). Pancreatic edema score, inflammation score, hemorrhage score, necrosis score as well as total score were elevated over time and peaked at 72h (**Figures 2C–G**). Moreover, the expression of TNF-α and IL-6, inflammatory mediators of acute inflammation, tended to increase over time and peaked at 72h, which was consistent with the trend of the pathological score (**Figures 2A, H, I**).

### CP Induced by Partial Ligation of Pancreatic Duct Can Simulate Clinical CP

**Figure 3:**
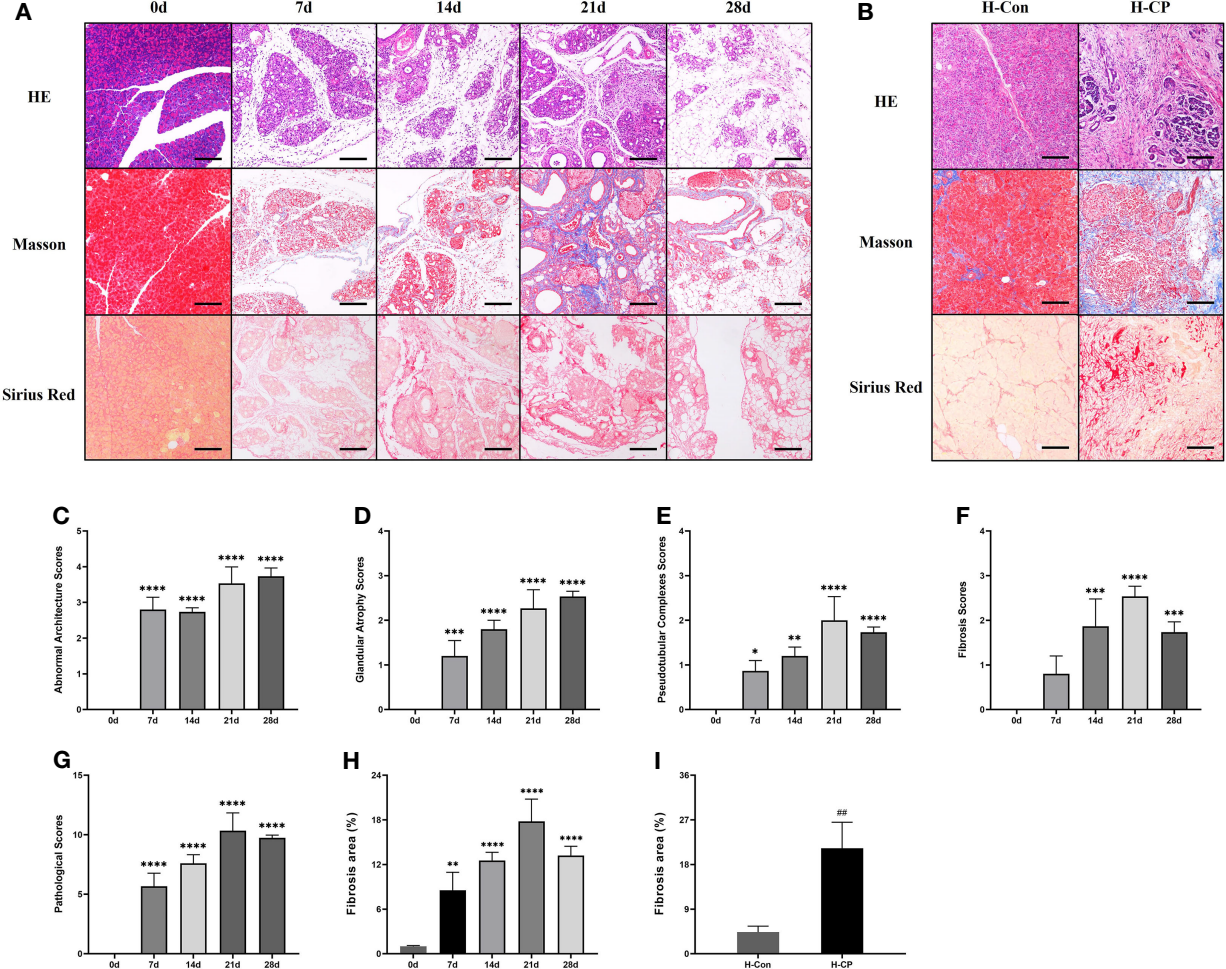
The pancreatic sections of CP mice model induced by ligation of the pancreatic duct showed progressively aggravated glandular atrophy, acinar cell damage, fibrosis, lipomatosis, as well as the formation of pseudobulbar complexes. The pancreatic specimens from CP patients also exhibited obvious fibrosis and parenchymal loss. **(A)** HE, Masson and Sirius Red staining of pancreas in mice at different time points. **(B)** HE, Masson and Sirius Red staining of human pancreatic specimens. **(C)** Pancreatic abnormal architecture scores in mice. **(D)** Pancreatic glandular atrophy scores in mice. **(E)** Pancreatic pseudobulbar complexes scores in mice. **(F)** Pancreatic fibrosis scores in mice. **(G)** Total pathological scores in mice. **(H)** Pancreatic fibrosis area in mice. **(I)** Pancreatic fibrosis area in human specimens. Values are shown as means ± SE. n = 3 per group. *P < 0.05, **P < 0.01, ***P < 0.001, ****P < 0.0001 versus 0d. ^##^p < 0.01 versus H-Con. Scale bar=150μm. H-CP, human chronic pancreatitis; H-Con, human control.

**Figure 4:**
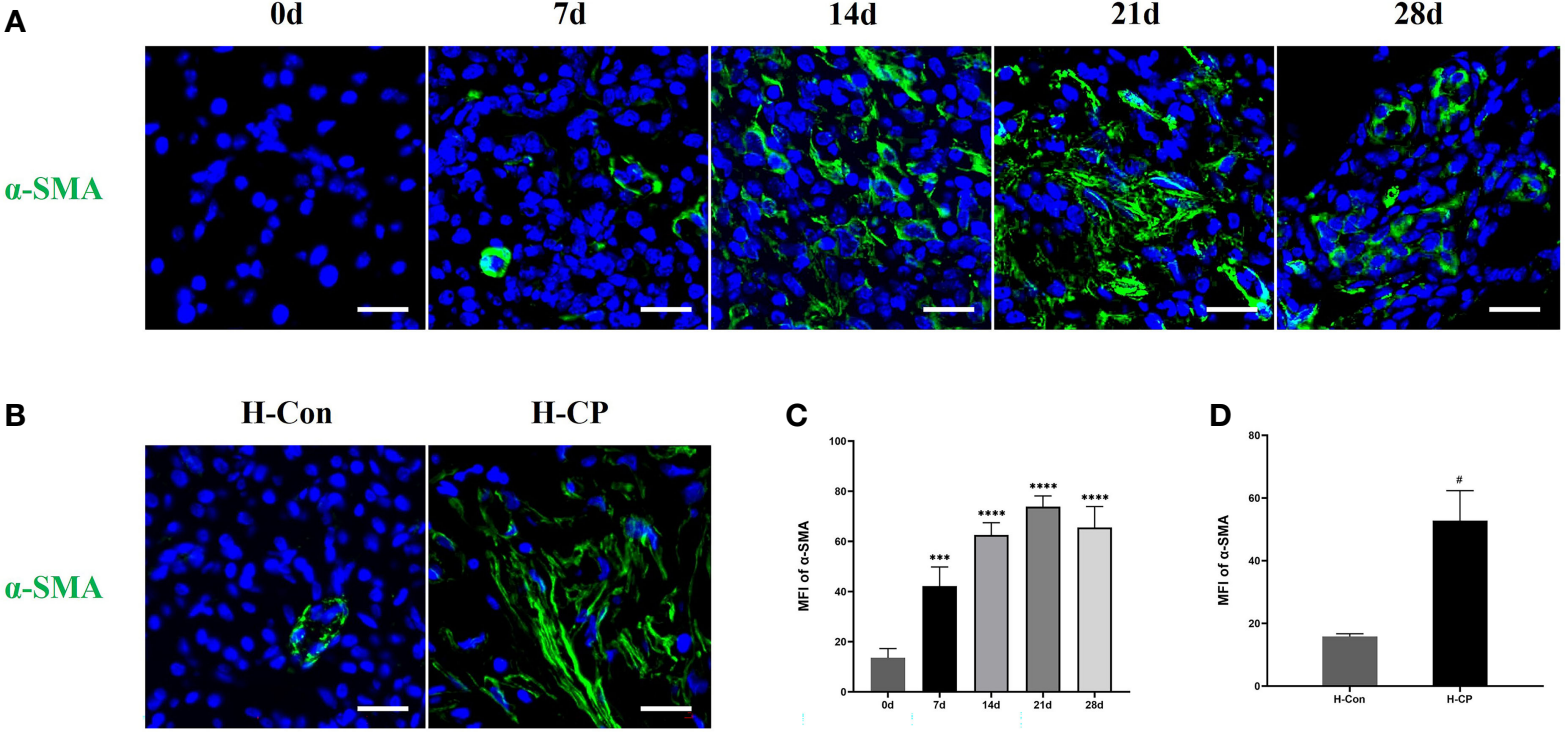
The expression of pancreatic α-SMA was significantly upregulated in CP mouse at 14d, 21d, and 28d post ligation and peaked at 21d, it was also upregulated in human CP specimens compared to normal tissue. **(A)** Immunofluorescence staining of pancreatic α-SMA in mice at different time points. **(B)**. Immunofluorescence staining of pancreatic α-SMA in human specimens. **(C)** MFI of pancreatic α-SMA at different time points in mice. **(D)** MFI of pancreatic α-SMA in human specimens. Values are shown as means ± SE. n = 3 per group. ***P < 0.001, ****P < 0.0001 versus 0d. ^#^P < 0.05 versus H-Con. Scale bar=20μm. H-CP, human chronic pancreatitis; H-Con, human control; MFI, mean fluorescence intensity.

After partial ligation of the pancreatic duct, the pancreatic sections of mice showed glandular atrophy, acinar cell damage, interstitial fibrosis, lipomatosis, as well as replacement of exocrine tubules by pseudobulbar complexes over time (**Figure 3A**). We evaluated CP injury based on the Demols criteria. The results showed that the abnormal architecture score and glandular atrophy score were elevated over time and reached the maximum at 28d (**Figures 3C, D**), while pseudobulbar complexes and fibrosis score were elevated over time and peaked at 21 days (**Figures 3E, F**). Eventually, the total pathological score of 21d was the highest (**Figure 3G**), suggesting that 21d was the time point when CP injury was the severest in our study. Masson and Sirius red staining showed that fibrosis appeared from 7d, peaked on 21d, and still existed at 28d. However, due to lipomatosis (26), the fibrotic area decreased compared with that at 21d (**Figures 3A, H**). We collected some pancreatic tissues from clinical CP patients with a background of obstructive etiologies and human nonmalignant pancreatic patients for the HE, Masson and Sirius red staining, which exhibited obvious fibrosis and parenchymal loss (**Figure 3B**), and the percentage of fibrosis area in human CP specimen was closer to that at 21d in mice (**Figures 3H, I**). α-SMA is a common index of fibrosis in pancreatitis (27), immunofluorescence staining showed that the expression of α-SMA was significantly upregulated in CP mouse at 14d, 21d, and 28d post ligation and peaked at 21d, which was consistent with the trend of Masson and Sirius red staining (**Figures 4A, C**). In addition, α-SMA expression was significantly upregulated in human CP specimens compared to normal tissue (**Figures 4B, D**). Therefore, we concluded that the lesions on 21d of the mouse CP model are similar to those of clinical CP and can well mimic clinical CP.

### Macrophage Polarization and Pyroptosis Are Involved in the Development of Pancreatitis

**Figure 5:**
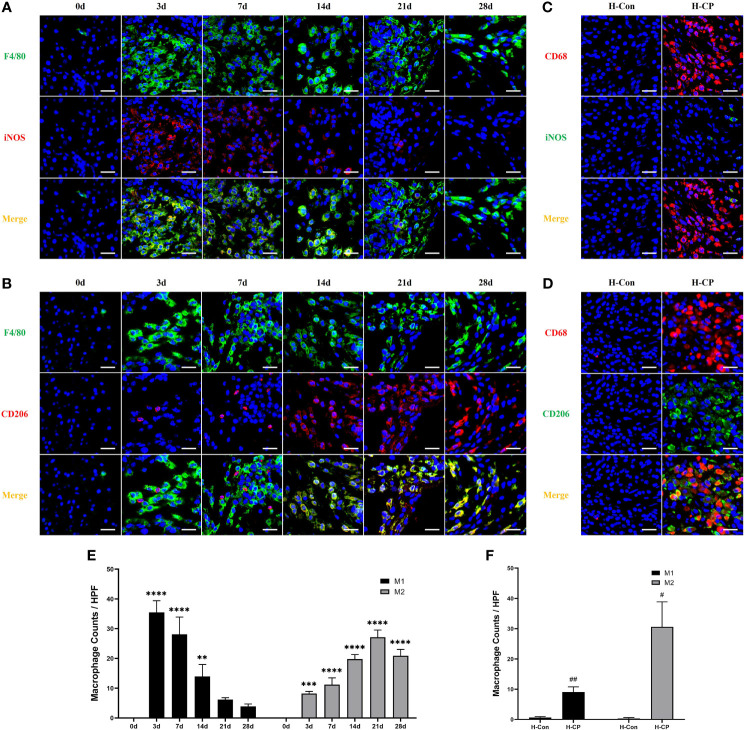
The subtype of macrophages changes when AP shifted to CP. In AP (3d) and the early phase of CP (7d), macrophages were predominantly M1 subtype (F4/80+iNOS+ cells). From 14d onwards, it was the M2 subtype (F4/80+CD206+ cells) of macrophages that was predominant, which was peaked at 21d. For human CP specimens, the pancreatic infiltrating macrophages were also predominantly M2 subtype (CD68+CD206+ cells) rather than M1 (CD68+iNOS+ cells) subtype. **(A, B)** Immunofluorescence staining of pancreatic infiltrating M1 and M2 macrophages in mice model of pancreatitis at different time points. **(C, D)** Immunofluorescence staining of pancreatic infiltrating M1 and M2 macrophages in human specimens. **(E)** Pancreatic M1 and M2 macrophage counts per HPF in mice. **(F)** Pancreatic M1 and M2 macrophage counts per HPF in human specimens. Values are shown as means ± SE. n = 3 per group. **P < 0.01, ***P < 0.001, ****P < 0.0001 versus 0d. ^#^p < 0.05, ^##^p < 0.01 versus H-Con. Scale bar=20μm. H-CP, human chronic pancreatitis; H-Con, human control; HPF, high-powered field of view.

A substantial of pancreatic infiltrating macrophages (F4/80+cells) were observed in both acute and chronic phases in mice with pancreatitis induced by partial pancreatic duct ligation. We found that macrophages were mainly distributed in the pancreatic interstitium at the early stage, whereas they began to migrate into the parenchyma as the disease course progressed. Only a few pancreatic resident macrophages were present in the pancreas of mice in the control group (**Supplementary Figure 2**), consistent with the previous report (28). The same pattern of macrophage (CD68+cells) infiltration and distribution was present in human pancreas specimens (**Supplementary Figure 3**). Macrophages are heterogeneous and their phenotype and functions are regulated by the surrounding microenvironment (29). Our results showed that in the AP model (3d), there was a large infiltration of macrophages in the pancreas and they mainly exhibited the M1 subtype with pro-inflammatory properties (**Figures 5A, B, E**). Notably, the subtype of macrophages started to change when AP shifted to CP. In the early phase of CP (7d), macrophages were still predominantly M1 subtype (F4/80+iNOS+ cells), but a certain amount of M2 macrophages (F4/80+CD206+ cells) had appeared. From 14d onwards, it was the M2 subtype of macrophages that was predominant, and the proportion of M2 macrophages peaked at 21d (**Figures 5A, B, E**). For human CP specimens, the pancreatic infiltrating macrophages were also predominantly M2 subtype (**Figures 5C, D, F**). Thus, the mouse CP model can also ideally mimic the macrophage profile of human CP.

**Figure 6:**
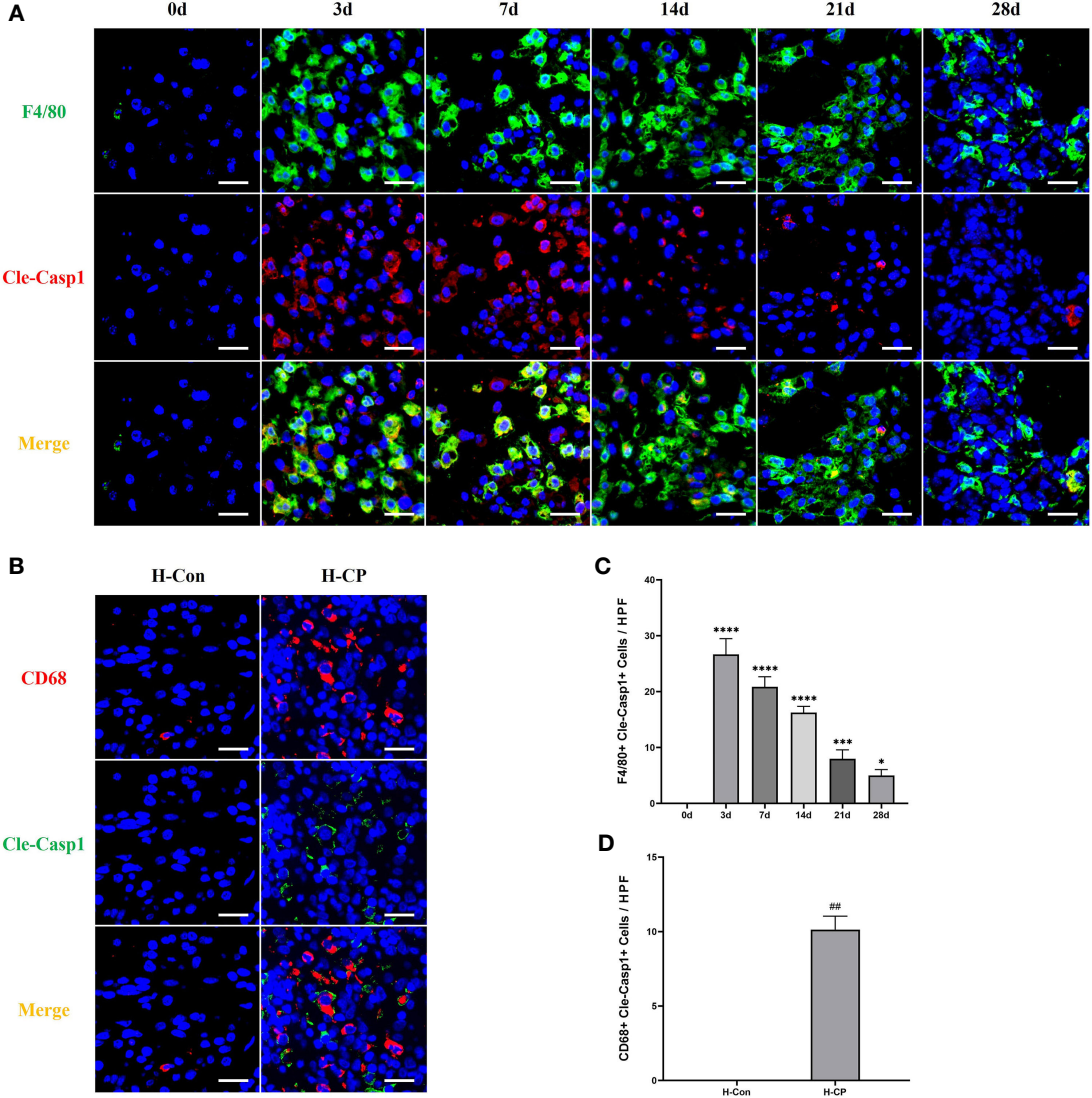
Pyroptotic macrophages (F4/80+Cleaved-Caspase1+cells) were observed more in AP, which decreased over time in CP. **(A)** Immunofluorescence staining of pancreatic pyroptotic macrophages in mice model of pancreatitis at different time points. **(B)** Immunofluorescence staining of pancreatic pyroptotic macrophages in human specimens. **(C)** Pancreatic pyroptotic macrophages counts per HPF in mice. **(D)** Pancreatic pyroptotic macrophages counts per HPF in human specimens. Values are shown as means ± SE. n = 3 per group. *P < 0.05, ***P < 0.001, ****P < 0.0001 versus 0d. ^##^p < 0.01 versus H-Con. Scale bar=20μm. H-CP, human chronic pancreatitis; H-Con, human control; HPF, high-powered field of view.

Macrophage pyroptosis is a strong promoter of inflammation, characterized by assembly of inflammasomes, activation of caspase-1 to cleaved caspase1, cleavage, and release of downstream IL-1β and IL-18 (29). Our results showed that macrophage pyroptosis (F4/80+Cleaved-Caspase1+ cells) was associated with the development of pancreatitis, however, it occurred more in AP and started to decrease over time in CP, which seemed to be similar to the trend in M1 macrophages in mouse CP models, as well as in human CP (**Figures 6A–D**).

### IL-4 Is Involved in the Development of CP and Is Mainly Derived From PSCs

**Figure 7:**
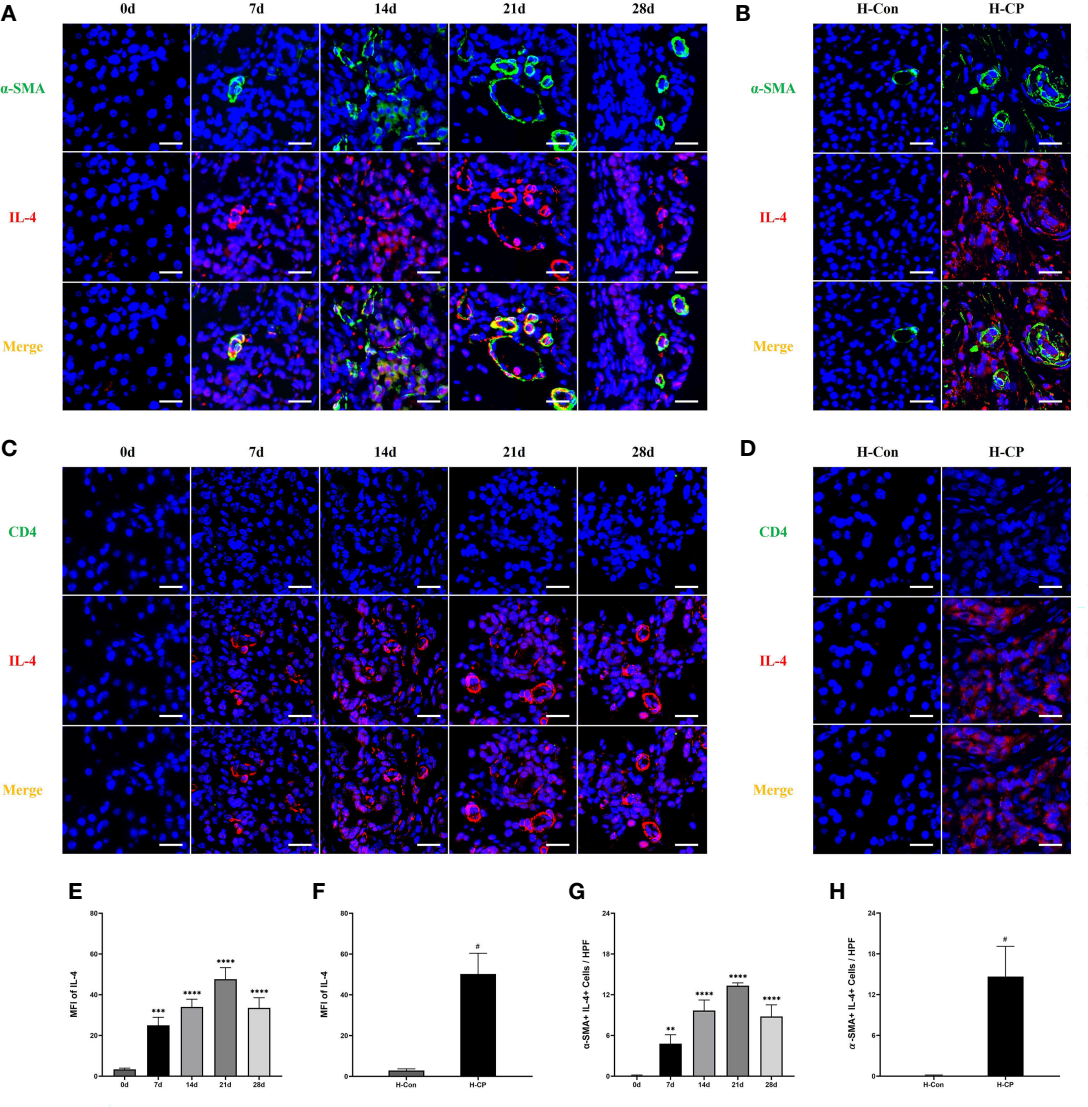
The expression of IL-4 increased in human CP specimens, as well as in mice CP models as the disease progressed and peaked at 21d. A large number of α-SMA+IL-4+cells, which represents PSCs secreting IL-4, were found in the pancreas of mice CP models and human CP. However, almost no CD4+IL-4+cells in the pancreas of mice CP models and human CP were observed. **(A, C)** Immunofluorescence staining of pancreatic IL-4 co-localized with α-SMA or CD4 in mice model of pancreatitis at different time points. **(B, D)** Immunofluorescence staining of pancreatic IL-4 co-localized with α-SMA or CD4 in human specimens. **(E)** MFI of pancreatic IL-4 at different time points in mice. **(F)** MFI of pancreatic IL-4 at different time points in human specimens. **(G)** α-SMA+IL-4+cells counts per HPF in mice. **(H)** α-SMA+IL-4+cells counts per HPF in human specimens. Values are shown as means ± SE. n = 3 per group. **P < 0.01, ***P < 0.001, ****P < 0.0001 versus 0d. ^#^P < 0.05 versus H-Con. Scale bar=20μm. H-CP, human chronic pancreatitis; H-Con, human control; MFI, mean fluorescence intensity; HPF, high-powered field of view.

IL-4, one of the members of the Th2 cytokine family, is important for macrophage polarization toward the M2 subtype (30), it has also been reported to be associated with pancreatic fibrosis (31). We explored IL-4 dynamics in mice CP models and human CP specimens. The results showed that the expression of IL-4 increased in mice CP models as the disease progressed and peaked at 21d (**Figures 7A, E**), which was consistent with the trend of fibrosis. There was also a significant expression of IL-4 in human CP specimens (**Figures 7B, F**). We further explored the source of pancreatic IL-4 in CP. PSCs are involved in the development of pancreatic fibrosis (32). In addition, it is reported that helper T cells (Th cells) are associated with the M2 polarization of macrophages (33). However, we only observed a small number of CD4+ cells in the pancreas of mice CP models and human CP, with little co-localization of CD4 and IL-4 (**Figures 7C, D**). Interestingly, the presence of a large number of α-SMA+IL-4+cells, which represents PSCs secreting IL-4, were found in the pancreas of mice CP models and human CP (**Figures 7A, B, G, H**). Therefore, we considered that PSCs are the main source of IL-4 both in murine CP and human CP, which is closely associated with pancreatic fibrosis.

## Discussion

Pancreatitis is an inflammatory disease, which begins with the premature activation of digestive enzymes in pancreatic acinar cells and leads to cell damage (7, 34). Most of the knowledge about the pathophysiology of this disease comes mainly from animal experiments. Drugs-induced AP models are mainstream research methods, the most commonly used drugs are cerulein, followed by L-arginine, which has the advantages of simplicity and high rate of success (35–37). Nevertheless, the correlation between these two models and human AP remains to be discussed. More importantly, the cerulein-induced AP model is mostly mild edematous pancreatitis with only a small amount of necrosis (38, 39), which is not suitable for simulating clinical severe acute pancreatitis (SAP). AP models constructed by specific diet or surgery in mice can often better simulate the etiology of human AP and has higher clinical transformation value, such as alcoholic AP model constructed by oral administration of ethanol and palmitoleic acid (40), alcoholic AP with a background of hypophosphatemia constructed by low-phosphate diet (LPD) feeding followed by orogastric administration of ethanol (41), hypertriglyceridemic AP induced by oral administration of high-fat diet (HFD) with or without intraperitoneal injection of cerulein (42, 43), biliary AP model constructed by retrograde injection of sodium taurocholate into the pancreaticobiliary duct (44), and the partial ligation of pancreatic duct proposed in recent years (13). However, the application of the surgical AP model in mice is still not common. In our study, we found that the serum amylase level of mice AP models elevated 4 hours after pancreatic duct ligation, suggesting pancreatic damage. The pancreatic injury gradually aggravated over time, as evidenced by elevated HE pathological scores and upregulated expression of IL-6 and TNF-α, and eventually reached the peak at 72h. In addition to the significant edema and inflammatory cell infiltration of the pancreas, there is also a large amount of acinar cell necrosis and hemorrhage, which is a significant difference from cerulein-induced AP (38, 39), and is more consistent with clinical SAP. Therefore, our model is suitable for the scientific research of SAP with a background in pancreatic ductal hypertension.

CP, as an irreversible fibrotic disease, is still challenging to study in-depth. Similar to the AP model in mice, repeated injection of cerulein is also the mainstream means to construct mouse CP with a high reliability and reproducibility. However, it has the disadvantage of low correlation with human CP (45). As a skill-dependent modality, pancreatic duct ligation is superior in simulating human CP caused by obstructive etiologies (46). We explored the morphological changes of the pancreas in 7, 14, 21, and 28 days after pancreatic duct ligation. The results showed that significant lesions appeared in the pancreas from 7d. With the passage of time, there were varying degrees of glandular atrophy, ductal dilatation, interstitial fibrosis, tissue proliferation, inflammatory cell infiltration, and fibrosis marker α-SMA expression. We considered that 21d was the time point when CP injury was the severest in our study based on Demols criteria. Interestingly, the pancreatic parenchyma was replaced by a large amount of adipose tissue at 28d after surgery, namely lipomatosis (26). In order to verify the correlation between the mouse CP model constructed by pancreatic duct ligation and human CP, we collected three clinical samples of patients with CP caused by obstructive etiologies and three normal pancreatic samples for research. The results showed that the status of mouse pancreas at 21d was similar to that of human CP with a background of obstructive etiologies in terms of pancreatic morphological changes and degree of fibrosis. Therefore, we recommend 21d as the best time point to study CP in this model.

It is now well established that immune cells are major contributors to the pathogenesis of pancreatitis, and among these, macrophages make up a key innate immune population (10). Studies have shown that in mice with cerulein-induced AP and CP, the infiltrating macrophages in the pancreas of the former are predominantly of the M1 subtype, whereas those of the latter are M2 (18–21). However, there is still a lack of evidence regarding macrophage status, their regulatory mechanisms, and relevance to human CP in murine pancreatitis models constructed by partial ligation of pancreatic ducts. In recent years, investigators have focused on macrophage pyroptosis, a caspase-1-dependent programmed cell death that culminates in cell lysis and release of mature IL-1β and IL-18 thereby aggravating the inflammatory response (47). Macrophage pyroptosis has been reported to play a role in the development of hepatitis (48), Alzheimer’s disease (49), AP (22, 50), etc. In our study, we found that there were a large number of M1 macrophages in the pancreas of AP mice constructed by partial ligation of the pancreatic duct, accompanied by a large number of F4/80+cleaved caspase1+ cells, which indicate macrophage pyroptosis. In CP mice constructed by partial ligation of the pancreatic duct, M1 and M2 macrophages in the pancreas went in opposite directions. At the early stage of CP (7 days after ligation), M1 macrophages were still far more numerous than the M2 subtype. The numbers of M1 and M2 macrophages were roughly the same at 14 days after ligation. Whereas by 21 days post ligation, the number of M2 macrophages had peaked, a time point when the macrophage polarization state is highly similar to human CP. Macrophage pyroptosis, as a pro-inflammatory cell death manner, occurred more in AP and the early stage of CP in our model, then started to decrease over time, which seemed to be similar to the trend of M1 macrophages in mice. For human CP specimens, fewer pyroptotic macrophages were observed. These results suggest that a mouse CP model constructed by partial ligation of the pancreatic duct for 21 days could ideally mimic the pattern of macrophage changes in human CP and be suitable for related studies.

It is of great clinical value to explore the mechanism of pancreatic fibrosis in CP. The role of PSCs has been studied with great interest (51). PSCs can be activated by chemokines released as a result of pancreatic inflammation and drive pancreatic parenchymal fibrosis (8). Up-regulated expression of α-SMA is the hallmark of PSCs activation (52, 53). In addition to producing collagen and extracellular matrix, PSCs were reported to mediate pancreatic fibrosis by modulating M2 macrophages in cerulein-induced CP in mice, and IL-4, as a member of the Th2 cytokine family, played a key role in this regulatory process (21). In our study, we found that in human CP, pancreatic expression of α-SMA and IL-4 was significantly upregulated. In the CP mice model constructed by partial ligation of the pancreatic duct, the expression of pancreatic α-SMA and IL-4 began to be upregulated 7 days after ligation and reached a peak by 21 days, consistent with the trend of M2 macrophages. More importantly, there was substantial immunofluorescence colocalization of α-SMA and IL-4, suggesting that PSCs are a major source of IL-4 in human CP and in murine CP constructed by partial ligation of the pancreatic duct. Next, we further explored whether Th2 cells were another source of IL-4 since Th2 cells were reported to be elevated in the peripheral blood of patients with CP (54) and could regulate M2 macrophage polarization in atherosclerosis (55). However, our results showed a low number of T cells infiltrating the pancreas in human CP and in CP mice model constructed by partial ligation of the pancreatic duct, as well as an almost absent immunofluorescent colocalization of CD4 and IL-4, suggesting that Th2 cells are not another source of IL-4. Therefore, we speculate that in CP caused by obstructive etiologies, Th2 cells hardly infiltrated into the pancreas, so they did not directly regulate the polarization of macrophages.

In conclusion, our study explored the pancreatic pathomorphology and immune microenvironment characteristics in AP and CP in a mouse model constructed by partial ligation of the pancreatic duct, as well as the relation between this model and human CP. Overall, the murine pancreatitis model constructed by partial ligation of the pancreatic duct, especially the CP model, is highly similar to human CP caused by obstructive etiologies in terms of morphological alterations and immune microenvironment characteristics and is recommended for further studies. However, it should be recognized that no model can fully share features of the human disease, it is still necessary to understand more deeply the pathophysiological mechanisms of different models and the advantages of different models in order to select a specific model for a specific purpose.

## Data Availability Statement

The original contributions presented in the study are included in the article/**Supplementary Material**. Further inquiries can be directed to the corresponding authors.

## Ethics Statement

The studies involving human participants were reviewed and approved by Ethics Committee of the Third Xiangya Hospital of Central South University. Written informed consent for participation was not required for this study in accordance with the national legislation and the institutional requirements. The animal study was reviewed and approved by Central South University institutional animal care and use committees.

## Author Contributions

CP and GT designed the experiment, analyzed data, and wrote the manuscript. CP and GT performed research. LY and PW assisted in completing the experiment, XZ and ZheL reviewed and modified the manuscript. ZhiL and XY provided overall guide and supervision. All authors contributed to the article and approved the submitted version.

## Funding

The current study was funded by the National Natural Science Foundation of China (No. 81873589). Natural Science Foundation of Hunan Province (No. 2020JJ4858). Science and technology planning project of Changsha (No. kq2004143).

## Conflict of Interest

The authors declare that the research was conducted in the absence of any commercial or financial relationships that could be construed as a potential conflict of interest.

## Publisher’s Note

All claims expressed in this article are solely those of the authors and do not necessarily represent those of their affiliated organizations, or those of the publisher, the editors and the reviewers. Any product that may be evaluated in this article, or claim that may be made by its manufacturer, is not guaranteed or endorsed by the publisher.

## Supplementary Material

Supplementary Figure 1 Intraoperative photograph of partial ligation of the pancreatic duct in mice.

Supplementary Figure 2 Immunofluorescence staining of pancreatic macrophages (F4/80+cells) in mice at different time points with magnification of the areas indicated by the white square. Scale bar=100μm.

Supplementary Figure 3 Immunofluorescence staining of pancreatic macrophages (CD68+cells) in human specimens with magnification of the areas indicated by the white square. Scale bar=100μm. H-CP, human chronic pancreatitis; H-Con, human control.
