# Supplementary figures and images for: Murine Chronic Pancreatitis Model Induced by Partial Ligation of the Pancreatic Duct Encapsulates the Profile of Macrophage in Human Chronic Pancreatitis
Source: Front Immunol. 2022 Apr 1;13:840887. doi: 10.3389/fimmu.2022.840887 (PMC9011002; doi:10.3389/fimmu.2022.840887)

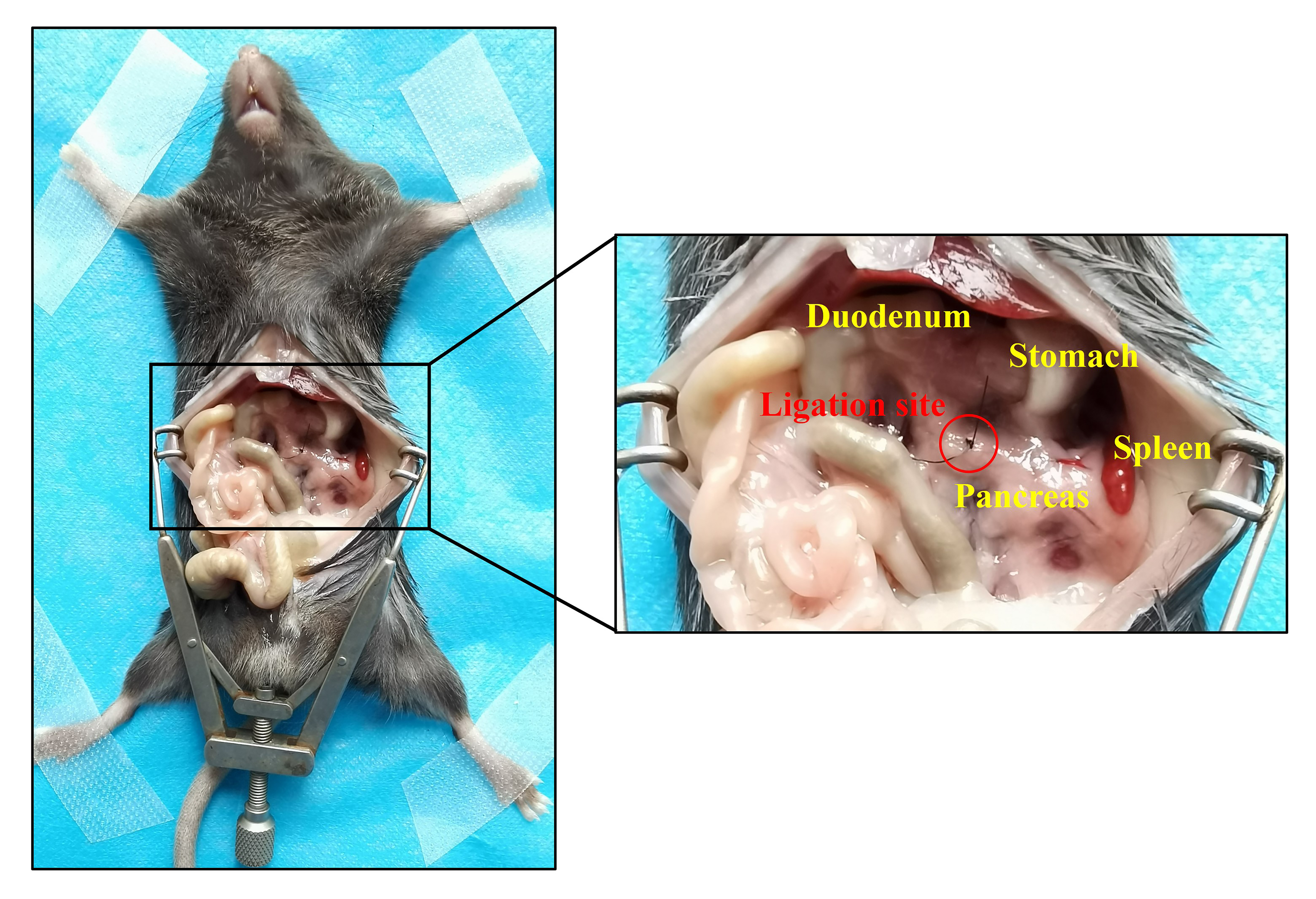

Supplement: Supplementary Figure 1 — Intraoperative photograph of partial ligation of the pancreatic duct in mice. [file Image_1.jpg]

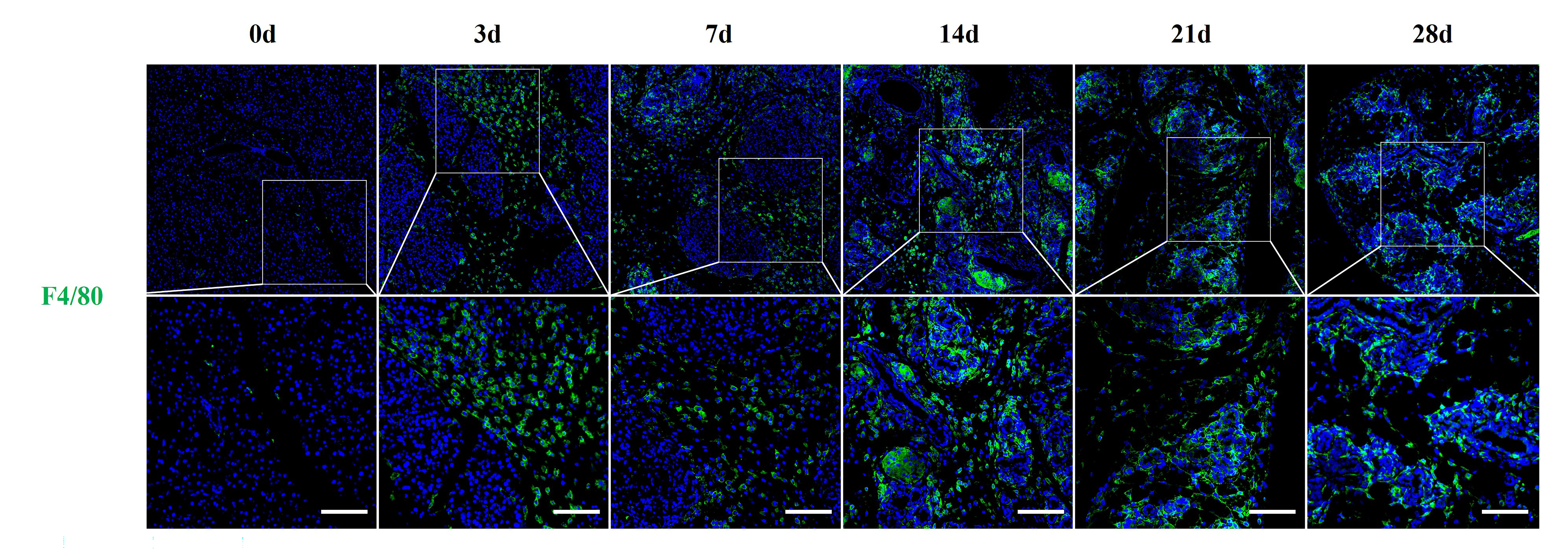

Supplement: Supplementary Figure 2 — Immunofluorescence staining of pancreatic macrophages (F4/80+cells) in mice at different time points with magnification of the areas indicated by the white square. Scale bar=100μm. [file Image_2.jpg]

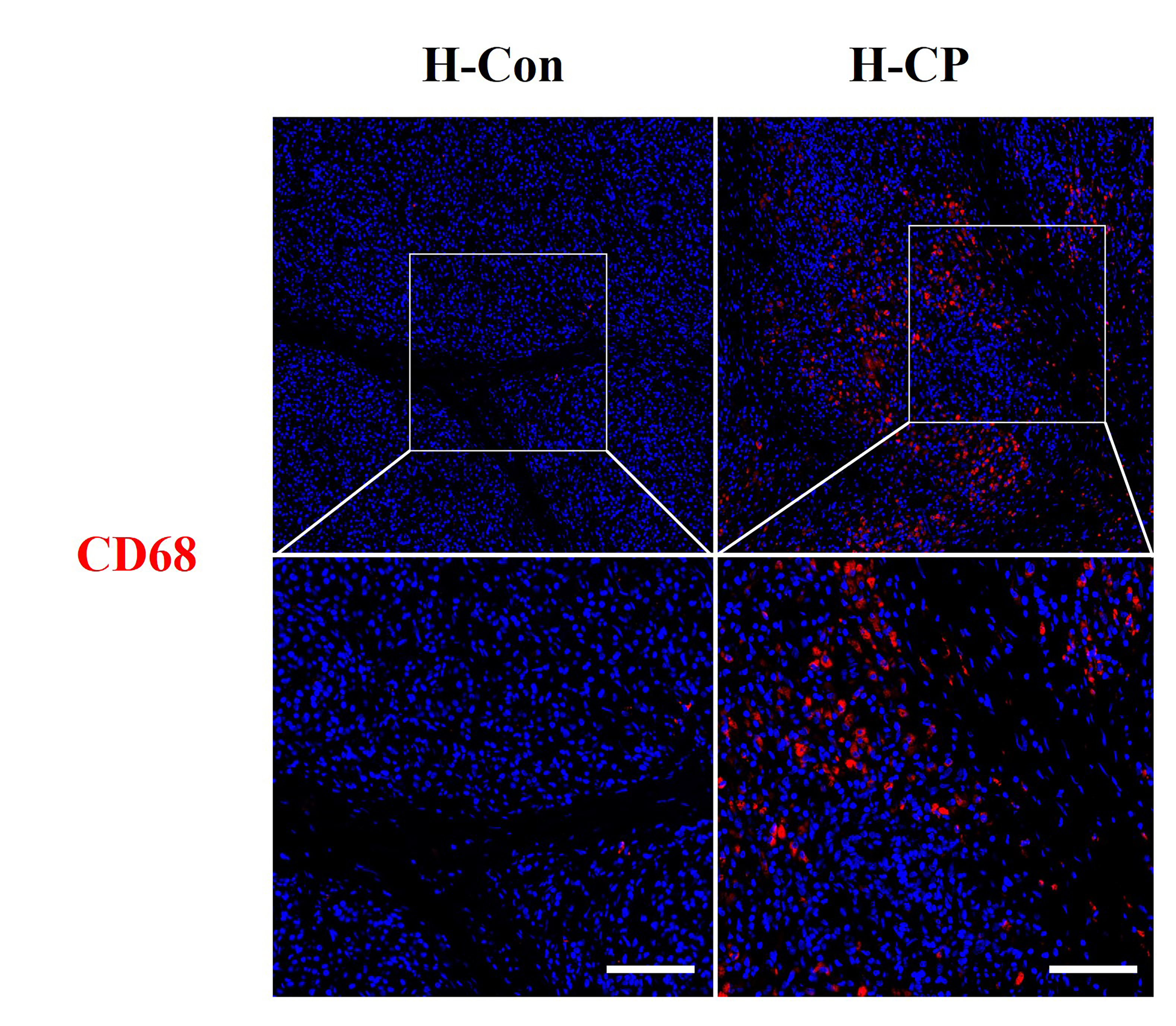

Supplement: Supplementary Figure 3 — Immunofluorescence staining of pancreatic macrophages (CD68+cells) in human specimens with magnification of the areas indicated by the white square. Scale bar=100μm. H-CP, human chronic pancreatitis; H-Con, human control. [file Image_3.jpg]
